# Synthesis, characterization, computational analyses, in silico ADMET studies, and inhibitory action against SARS-CoV-2 main protease (Mpro) of a Schiff base

**DOI:** 10.55730/1300-0527.3460

**Published:** 2022-06-02

**Authors:** Songül ŞAHİN, Necmi DEGE

**Affiliations:** 1 Department of Chemistry, Faculty of Art and Sciences, Ondokuz Mayıs University, Samsun, Turkey; 2 Department of Physics, Faculty of Art and Sciences, Ondokuz Mayıs University, Samsun, Turkey

**Keywords:** Schiff base, SARS-CoV-2, ADMET, X-ray, COVID-19, main protease (Mpro)

## Abstract

COVID-19 disease caused by the severe acute respiratory syndrome coronavirus (SARS-CoV-2) has struck the whole world and raised severe health, economic, and social problems. Many scientists struggled to find a vaccine or an antiviral drug. Eventually, both vaccines and recommended drugs, repurposed drugs, or drug combinations were found, but new strains of SARS-CoV-2 continue to threaten human life and health. As part of the fight against COVID-19 disease, this study involves an in silico molecular docking analysis on the main protease (Mpro) of SARS-CoV-2. To this aim, a Schiff base compound was synthesized and characterized using spectroscopic techniques, including X-ray, FTIR, and UV-Vis. Surface analysis and electronic properties of this molecule were investigated using the DFT method. The drug-likeness parameters of the title compound were studied according to the rules of Lipinski, Veber, Ghose, Egan, and Muegge and were found in agreement with these rules. In silico toxicity analyses revealed that the new compound is a potentially mutagenic and carcinogenic chemical. The title compound was predicted to be an inhibitor of cytochrome P450 enzymes (5 CYPs). This inhibitory effect indicates a weak metabolism of the molecule in the liver. In addition, this compound was displayed good intestinal absorption and blood-brain barrier penetration. The druggability properties of the title compound were investigated, and SwissTargetPrediction predicted it to be a protease inhibitor. In this context, the SARS-CoV-2 main protease was selected as a biological target in molecular docking studies. Docking results were compared with the known native ligand N3 inhibitor. The value of binding energy between the Schiff base compound and the binding pocket of the main protease is higher than that of the reference ligand N3. The calculated free energies of binding of the Schiff base compound and the reference ligand N3 are −8.10 and −7.11 kcal/mol, respectively.

## 1. Introduction

So far, seven coronaviruses, including HCoV-229E, HCoV-NL63, HCoV-OC43, HCoV-HKU1, SARS-CoV, MERS-CoV, and SARS-CoV-2, have been detected in humans. In late December 2019, COVID-19 disease related to the latest one, SARS-CoV-2, was reported for the first time in Wuhan, China [1,2]. The novel virus of 2019 was initially named 2019-nCoV by WHO. On February 11, 2020, the International Committee on Taxonomy of Viruses renamed the virus as severe acute respiratory syndrome coronavirus 2 (SARS-CoV-2) based on phylogenetic analysis [3]. In March 2020, the disease was declared a global pandemic because it has spread to over 100 countries [4]. As of December 4, 2021, the most recent data show that SARS-CoV-2 has caused 263,563,622 cases and 5,232,562 deaths worldwide [5] (Access date: December 04, 2021).

Clinical symptoms in pediatrics are usually absent or mild [6]. In adults, symptoms can range from moderate symptoms to severe health problems such as cough, fever, headache [7–10], dyspnea [8–10], myalgia [8, 9], tiredness, loss of taste or smell, aches, cold, discoloration of fingers or toes, breathing difficulties to dysfunction of organs [7], pneumonia [8, 9], acute respiratory distress syndrome (ARD), kidney failure, coagulation abnormalities, sepsis [9], multiple organ failure [8], cytokine storm [11]. At the beginning of the pandemic, when there was no FDA-approved vaccine, using various repurposed drugs or their combinations, including chloroquine, remdesivir [12–14], ribavirin [12, 13], galidesivir, tenofovir, sofosbuvir [12], favipiravir, lopinavir, hydroxychloroquine, oseltamivir, arbidol, interferons, ritonavir [13,14] methylprednisolone, bevacizumab, human immunoglobulin [14], darunavir, cefpiramide, tocilizumab [13] was recommended for the treatment of COVID-19. On August 23, 2021, the first COVID-19 vaccine, Pfizer-BioNTech’s vaccine, received full FDA approval [14].

The main protease (Mpro) of SARS-CoV-2, also known as 3C-like protease (3CLpro) [15], plays a pivotal role in viral replication and maturation, and has been reported as a valuable target for drug development against COVID-19 [16,17]. The monomeric structure of Mpro consists of 306 amino acids and three domains, including N-terminal domain-I (8–101 residues), N-terminal domain-II (102–184 residues), and C-terminal domain-III (201–306 residues) [16, 18]. HIS 41 and CYS 145 are catalytic dyads in the main protease [16, 19, 20], and they play an important role in protease activity [20, 21]. Therefore, inhibition of the catalytic dyad in 3CLPro may be a rational target for anti-CoV drug development [22].

Some authors have examined the inhibitory effect of Schiff base ligands on SARS-CoV-2 main protease (Mpro) using molecular docking methods [19, 23–26]. They found the binding energy of Mpro-ligand complexes between −5.88 and −9.10 kcal/mol. In this study, a new imine compound was synthesized. The molecular structure was determined by spectroscopic methods, including X-ray, FTIR, and UV-Vis. The electronic properties, surface characterization, and intermolecular interactions were calculated using the DFT and other techniques. In silico drug-like nature of the title compound was determined using the online in silico web tools. Druggability studies indicated that the title compound is most likely a protease inhibitor. In this sense, the SARS-CoV-2 main protease (Mpro) was selected as a biological target for the title compound in molecular docking experiments. Docking studies showed that the title compound had higher binding energy (−8.10 kcal/mol) against SARS-CoV-2 than the native N3 inhibitor (−7.11 kcal/mol).

## 2. Experimental

### 2.1. Materials and method

*Chemicals:* 5-nitro-2-(piperidin-1-yl)benzaldehyde; 2-methoxyaniline; ethanol/Sigma Aldrich.

*Apparatus*: Merck TLC plates/thin layer chromatography; CAMAG-UV cabinet/visualization; Stuart SMP 30/melting point; Precisa balance/weighing; Heidolph magnetic stirrer/heating.

*Spectrometers*: Perkin-Elmer/FT-IR; Thermo Fisher Scientific/UV-Vis; Stoe IPDS II [27]/X-ray.

*X-ray and computational analysis*: SHELXT [28], SHELXL [29]/solving and refinement; PublCIF [30]/CIF file; Gaussian 03 [31]/DFT [32] and B3LYP/6-31G (d, p) level of theory [33, 34]/frontier molecular orbitals, electrostatic potential map, Mulliken charges, geometrical parameters; Crystal Explorer [35]/Hirshfeld surfaces and fingerprint plots; Mercury [36]/crystal packing and intermolecular interactions.

*Docking and druglike nature:* AutoDock4 and AutoDockTools4 [37]/molecular docking; PDB [38]/3D structure of protein-ligand complex; PLIP [39]/secondary interactions and species; SwissADME [40]/pharmacokinetics, drug-likeness, and medicinal chemistry; PPB [41]/multitarget identification; ProTox-II [42] and pkCSM [43]/toxicity properties.

### 2.2. Synthesis

**Scheme 1.**
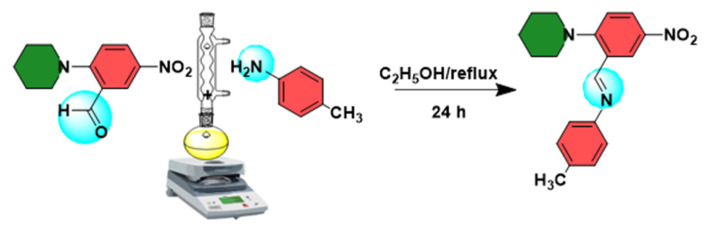
The synthesis reaction of the title compound.

The synthesis reaction of the (E)-N-(2-methoxyphenyl)-1-(5-nitro-2-(piperidin-1-yl)phenyl)methanimine compound is given in Scheme 1. First, a mixture of 5-nitro-2-(piperidin-1-yl)benzaldehyde (11 mg, 0.047 mmol) and 2-methoxyaniline (5.8 mg, 0.047 mmol) was dissolved in ethanol (25 mL) and the reaction temperature of the mixture was raised to the reflux temperature. The mixture was periodically analyzed by the thin-layer chromatography (TLC) using hexane: ethyl acetate (95:5) mobile phase to understand whether the reaction was completed. After the reactant plots disappeared in TLC, 22 h later, the reaction was completed. The solution was filtered and cooled at room temperature. The solvent was vaporized by the slow evaporation method in eight days. The yellow crystals were used for the whole analysis process. Melting point: 141–143 ºC. Yield: 85% (9.35 mg). C_19_H_21_N_3_O_3_. Molecular weight: 339.39 g/mol. FTIR (attenuated total reflectance, ATR), ν/cm^−1^: 3099, 3079 (Ar C-H); 2986, 2968, 2941, (Al C-H); 2900 (CH=N); 1681 (C=N); 1675, 1596, 1499, 1481 (Ar C=C); 1500, 1324 (NO_2_); 1248, 1232, 1063 (C-N); 1126, 1074 (C-O); 1025, 943, 910, 855, 828, 817, 765, 752, 715, 639, 517 (Figure S1). UV-Vis (in EtOH, 1.57 x E-04 M), λmax nm (logɛ): 361 (3.86) (Figure S2).

## 3. Results and discussion

### 3.1. Crystallographic, structural and geometrical parameters

**Figure 1:**
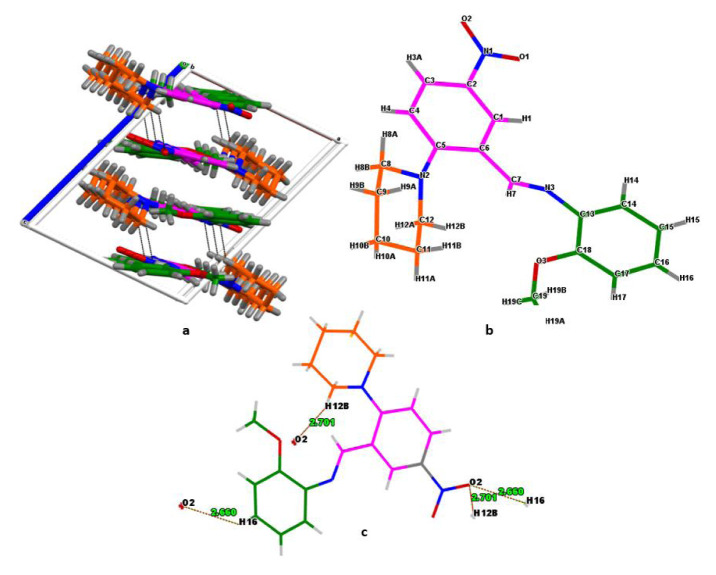
The unit cell viewing (**a**) numbering of the atoms in capped stick model (**b**) the secondary intermolecular interaction places and bond lengths (**c**) of the title compound.

Single-crystal X-ray diffraction data for the synthesized compound were collected using a STOE IPDS II diffractometer. Graphite monochromated MoKα radiation (λ = 0.7073 Å) was used for the measurements. The X-RED32, SHELXT, and SHELXL programs were used for cell refinement, structure solving, and refinement, respectively. The title compound with a molecular formula C_19_H_21_N_3_O_3_ and name (E)-N-(2-methoxyphenyl)-1-(5-nitro-2-(piperidin-1-yl)phenyl)methanimine is a small molecule with azomethine structure, yellow color, and prism-shape. It has space group P21/n and is crystallized in a monoclinic crystal system. As expected from this crystal system, three axes in the unit cell have unequal lengths: a ≠ b ≠ c (Å) = 10.1685, 13.3628, 13.416. The unit cell contains four monomeric molecules (Figure 1a). The symmetry operators of the crystal show that the structure is centrosymmetric; consequently, it is a nonlinear optical (NLO) inactive material. The geometric dimensions of the crystal selected for structure determination are 0.71 × 0.48 × 0.24 mm^3^. More information on the X-ray analysis and structural parameters can be seen in Table S1, which also lists the other crystal parameters. When we examine the molecule for its structural composition, we can identify four different molecular groups: anisole (green ring and -OCH_3_), C_7_H_7_O; nitrobenzene (purple ring and -NO_2_), C_6_H_3_NO_2_; piperidinyl (orange ring), C_5_H_10_N; and azomethine (cyan circle), CH=N. The azomethine group in the structure was bonded to the benzene rings, which have electron-withdrawing (NO_2_) and electron-donating (OCH_3_) substituents (Figure 1b and 1c). These substituents have a positive effect on the synthesis reaction by facilitating the condensation between amine and aldehyde reactants (through inductive and mesomeric effects). The calculated and measured bond lengths, bond angles, and dihedral angles are listed in Tables S2–S4, respectively. We have listed some important values here. Bond lengths (measured-calculated max/min.): Ar. C=C: 1.427–1.425 (C6-C5)/1.378–1.386 (C4-C3); Al. C-C: 1.517–1.530 (C12-C11)/1.529–1.533 (C11-C10); C-N: 1.479–1.478 (C12-N2)/1.406 (C5-N2)–1.400 (C13-N3); C7=N3: 1.277/1.282; 01-N1: 1.235/1.231; N1-O2: 1.230/1.233; C-O: 1.429–1.421 (C19-O3)/1.382–1.371 (C18-O3). Bond angles (measured/calculated): O1-N1-O2: 117.6/117.9; C7-N3-C13: 123.9/123.5; C6-C7-N3: 119.7/120.3; C1-C2-C3: 121.3/121.3; C9-C10-C11: 109.0/110.1.

### 3.2. Molecular electrostatic potential and Mulliken atomic charges

**Figure 2:**
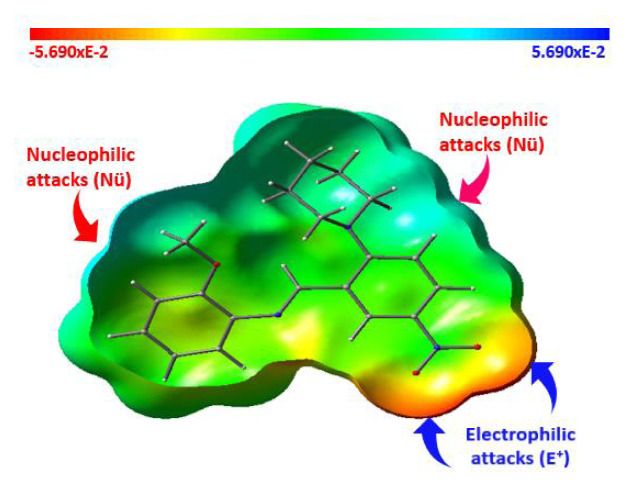
Molecular electrostatic potential map of the title compound.

**Figure 3:**
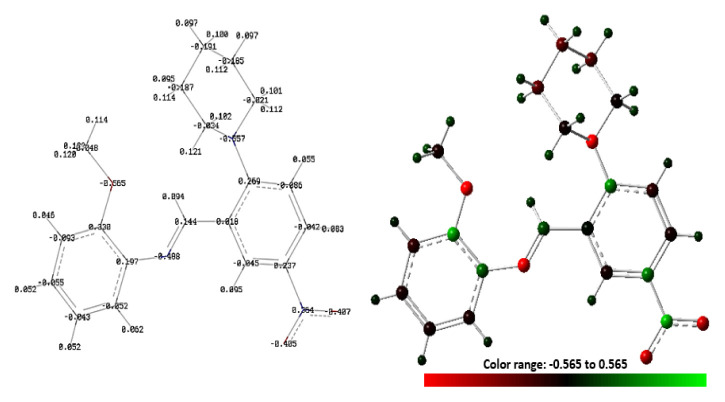
Mulliken atomic charge distribution map of the title compound: with numbered style (left), with colored style (right).

Molecular electrostatic potential (MEP) is a 3D mapping of the whole electron density in a molecule. It is a powerful method for determining the nucleophilic and electrophilic attack regions of a molecule. In MEP, the distribution of electrons is represented by colors. The color distribution can change in a red-blue gradient. The red regions show the most negative electrostatic potential. The blue regions show the most positive, and the green regions show the zero electrostatic potential in a molecule. While the red atoms, group of atoms, or regions are open to electrophilic attack, the blue regions are open to nucleophilic attack. The MEP map for our compound was calculated at the B3LYP/6-31G (d, p) level of theory and shown in Figure 2 with the three mapping models. As can be seen in Figure 2, the red regions spread over the oxygen atoms of the nitro group, and there are no dark blue regions showing strong nucleophilic attack positions in the molecule. The cyan regions were slightly gathered on the piperidinyl (C_6_H_5_N) ring and methyl (-CH_3_) group, and therefore, these regions can be evaluated as weak nucleophilic attack positions, while the oxygens atoms of the nitro group can be evaluated as strong electrophilic positions. The other positions of the molecule are green, have zero electrostatic potential; thus, these regions are not reactive. When the Mulliken charges of the title compound (Figure 3) were examined, the five atoms with the most negative and positive charges can be listed as follows: O3 (−0.565) > N2 (−0.557) > N3 (−0.488) > O2 (−0.407) > O1 (−0.405) and N1 (0.364) > C18 (0.330) > C5 (0.269) > C2 (0.237) > C13 (0.197).

### 3.3. Molecular orbital analysis and global reactivity descriptors

**Figure 4:**
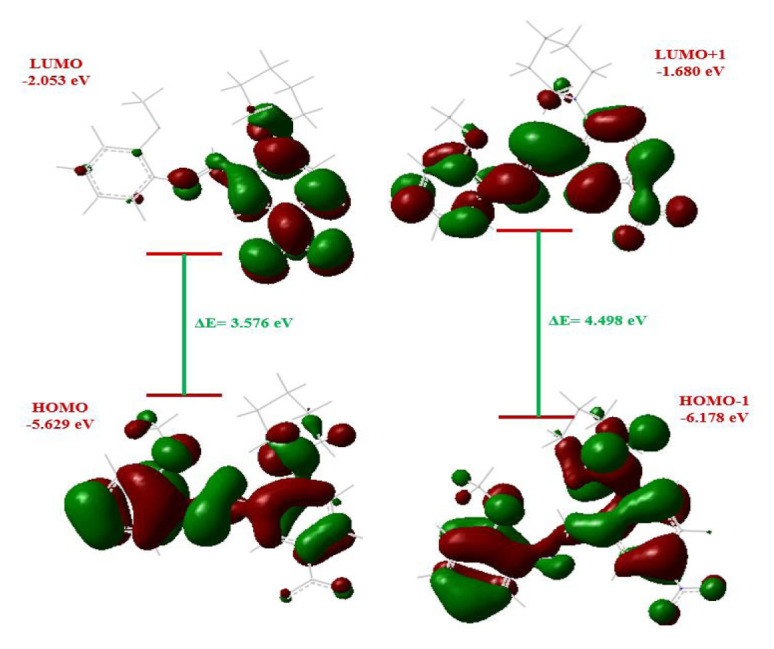
HOMO, HOMO-1, LUMO, LUMO+1 orbitals, energy values of the orbitals and band gaps of the title compound.

The highest occupied molecular orbital (HOMO) and the lowest unoccupied molecular orbital (LUMO), also called frontier molecular orbitals (FMOs), are the most critical orbitals in a molecule. The HOMO is an electron donor, while the LUMO is an electron acceptor. The energy of these orbitals and frontier orbital gap (or energy gap) plays a vital role in determining the chemical reactivity, kinetic stability [44–46], and electrical, optical, and physical properties [47] of a molecule. The global reactivity descriptors, including electron affinity, chemical hardness and softness, electronegativity, chemical potential, electrophilicity index, and charge transfer index can also be calculated using the energy values of these orbitals. The small energy gap between HOMO and LUMO indicates high chemical reactivity, low stability, softness [48], and easy charge transfer [49]; the large energy gap indicates low chemical reactivity, high kinetic stability [50], hardness [51], and hard charge transfer [49]. To investigate the above properties, we examined the FMOs of the compound in the gas phase. The calculations were performed at the B3LYP/6-31G (d, p) level of theory. HOMO-LUMO orbitals of the studied compound are shown in Figure 4. HOMO orbitals spread over all surfaces of the molecule except for some methylene groups in the piperidinyl ring; LUMO orbitals spread over imine, nitrobenzene, and nitrogen in the piperidinyl ring. Global reactivity descriptors were calculated using the HOMO and LUMO energies and are listed in Table 1. For the title molecule, the energy values of the HOMO, the LUMO, and the energy gap were found to be −5.629, −2.053, and 3.576 eV, respectively. Other global reactivity parameters can be seen in Table 1. The molecule has the smallest value of the energy gap among the similar studies we have performed previously [52–54]. From the calculated results, we can draw these conclusions about our compound: (i) The lowest value of the frontier orbital gap of the title molecule among the similar compounds [52–54] indicates that the molecule has high chemical reactivity, low chemical and kinetic stability, easy charge transfer, high polarizability, and softness. (ii) A high electrophilicity index (4.125) indicates a high electrophilic nature and good biological activity, while a relatively high softness value (0.279) indicates an increased probability of toxicity [55]. (iii) The effect of HOMO, LUMO, and energy gap values of the compounds on biological activity has been reported [56]. Low values of energy gap and LUMO lead to increased biological activity. This is due to the low energy required for electronic excitation and the strong charge transfer interaction between donor and acceptor atoms [57–59].

### 3.4. Hirshfeld surfaces and fingerprint analysis

**Figure 5:**
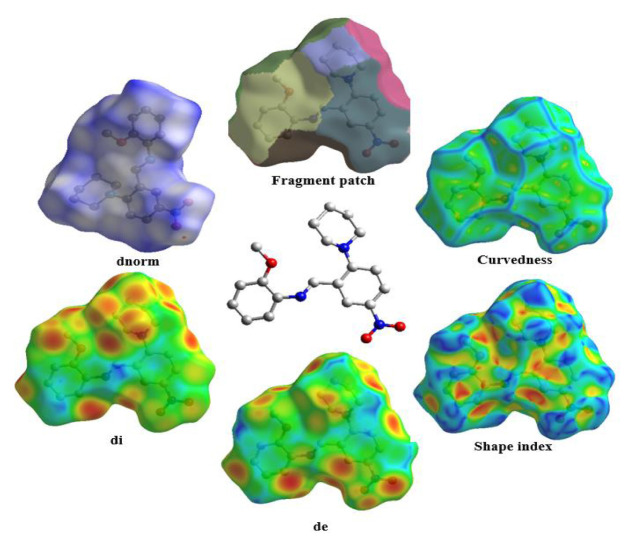
The title compound (middle) and its Hirshfeld surfaces (around).

**Figure 6:**
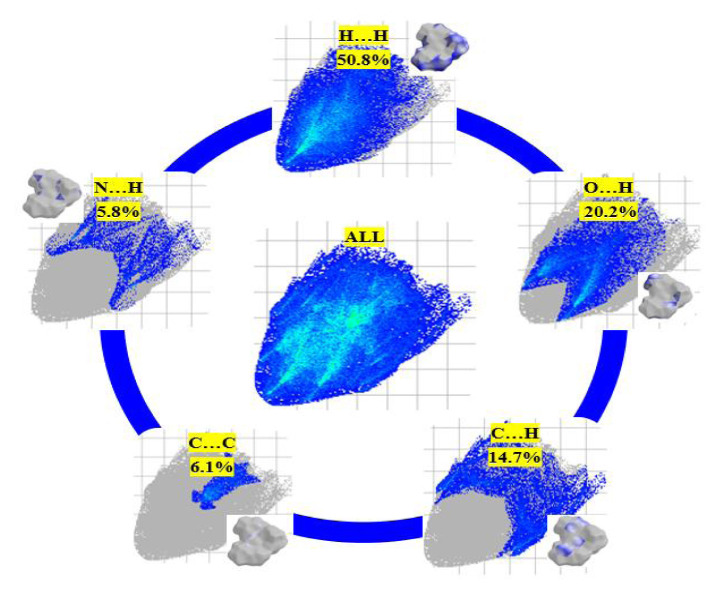
Intermolecular interactions and their relative contributions with the fingerprint plots (showed only bigger than 1% ones).

To learn about the intermolecular interaction species in the molecule and their quantitative contributions, we performed Hirshfeld surface and 2D fingerprint analyses. For the above purpose, Crystal Explorer 17.5 software requires the CIF file of the compound. The calculated Hirshfeld surfaces of the title compound are shown in Figure 5 in six different maps, including dnorm, di, de, fragment patch, curvedness, and shape index. In the dnorm mapped surface, red dots show the interaction distance shorter than the sum van der Waals (vdW) radii of two atoms. White regions show the close interactions to the vdW radii, and finally, the more extended contacts from the sum of vdW radii are represented by blue areas [60]. On the dnorm surface, the bright red spot is on the O2 atom, indicating the presence of hydrogen bonding at this atom. Fingerprint plots showing the percentage contributions of each interaction type are shown in Figure 6 (If the contribution is greater than or equal to 1%). The largest contribution to the percentage of secondary interactions is the hydrogen···hydrogen (50.8%) interaction, a type of vdW force. The second largest contribution is the hydrogen bonding interactions between oxygen and hydrogen (20.2%). The other contributors are C···H (14.7%), C···C (6.1%), and N···H (5.8%).

### 3.5. Docking studies

**Figure 7:**
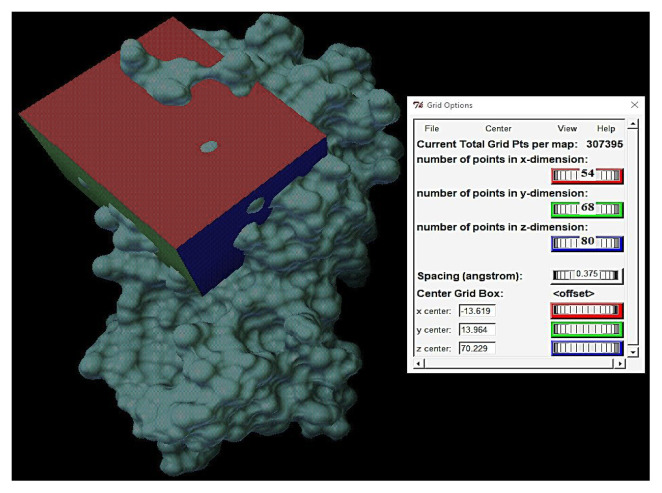
The active sites in the grid box and grid box settings.

Docking experiments were performed on the active residues of the SARS-CoV-2 main protease to determine the inhibitory activity of the synthesized compound. The 3D crystal structure of the main protease with the native N3 inhibitor (PDB entry: 6LU7 [61]) was retrieved from the RSCB PDB website (https://www.rcsb.org/). Before the docking experiments, the protein and ligand structures were prepared by removing water, adding polar hydrogens, merging nonpolar hydrogen atoms, and adding charges using the AutoDock and Autodock tools. The grid box has centered on the active residues [62], and the grid dimensions are given in Figures 7 and S3. The docking experiments were performed using the Lamarckian genetic algorithm. In the docking experiments, we used a semiflexible docking method (rigid target/flexible ligand).

**Figure 8:**
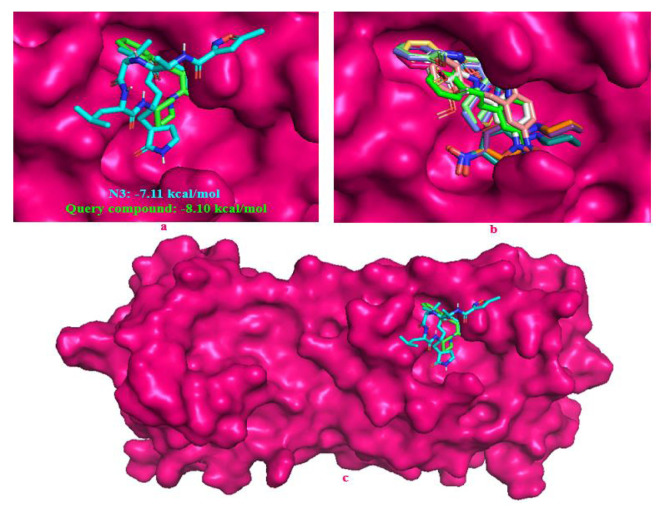
Docking positions of the reference N3 and query compound (**a**), ten positions produced of query compound in the active site of Mpro (**b**), the whole surface viewing of Mpro (**c**).

**Figure 9:**
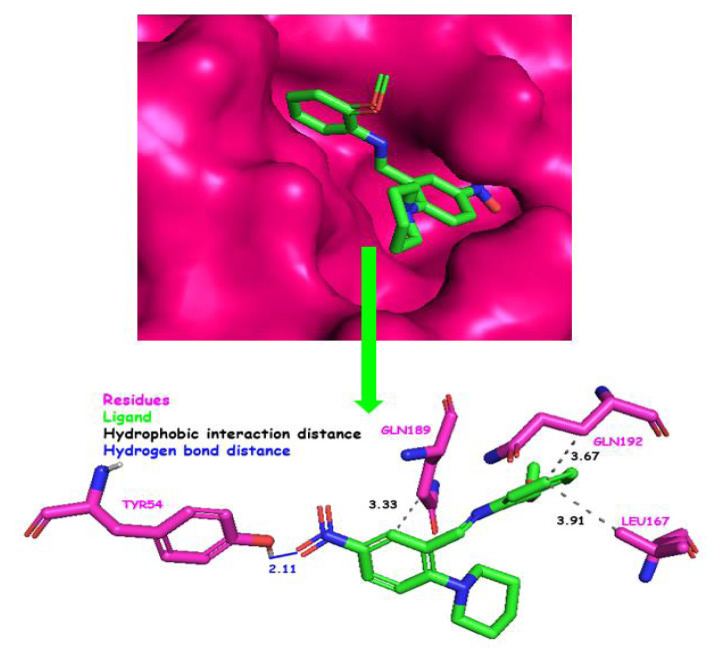
Docking position of the title compound in the active site of Mpro, and the secondary interaction species formed with the active residues.

N3 is a peptidomimetic inhibitor of the main protease of SARS-CoV and SARS-CoV-2 [61,63]. It was used for comparison in this study. Both the native ligand N3 inhibitor and query compound were docked to the active sites of the target protein. Docking results of the title compound, including binding modes, interacting residues, and binding free energy, are given in Figure 7. The docking experiment was repeated ten times for the title compound. Docking scores were determined with a standard deviation of 0.057. The median value of the docking experiments was determined and accepted as the final docking score (−8.10 kcal/mol). The ligand efficiency was found to be −0.32. The calculated docking parameters for ten docking experiments are shown in Figure S4. The most stable conformations of the reference N3 inhibitor and query compound (Figure 8a), the top ten conformations produced in the active pocket of SARS-CoV-2 main protease (Figure 8b), and the whole protein surface, including the reference molecule and query compound (Figure 8c), were shown in Figure 8. According to the docking results, the query compound has higher binding energy than the reference N3 inhibitor. In our study, the binding energies of the native ligand N3 and the query compound were calculated to be −7.11 and −8.10 kcal/mol, respectively. Query compound is bound to the target protein via both hydrophobic interactions (LEU167: 3.91 Å, GLN192: 3.67 Å, GLN189: 3.33 Å) and hydrogen bond (TYR54: 2.11 Å) (Figure 9). We compared the results of our study with those of the other studies (Table 2). For Mpro (3CLpro) of SARS-CoV-2, the studies listed in Table 2 show the chemical class studied against Mpro, common functional groups with our compound, software, docking scores, and interaction status with the catalytic dyad of Mpro. Although our docking score is high, no interaction with the catalytic dyad of Mpro was detected in our complex interactions. Therefore, the title compound may not provide the desired inhibitory effect on the Mpro of SARS-CoV-2.

### 3.6. Druglike nature, medicinal chemistry and druggability

**Figure 10:**
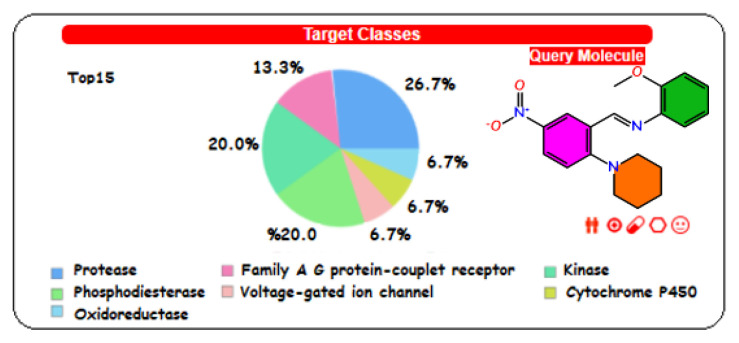
Druggability prediction results supplied from SwissTargetPrediction tool.

**Figure 11:**
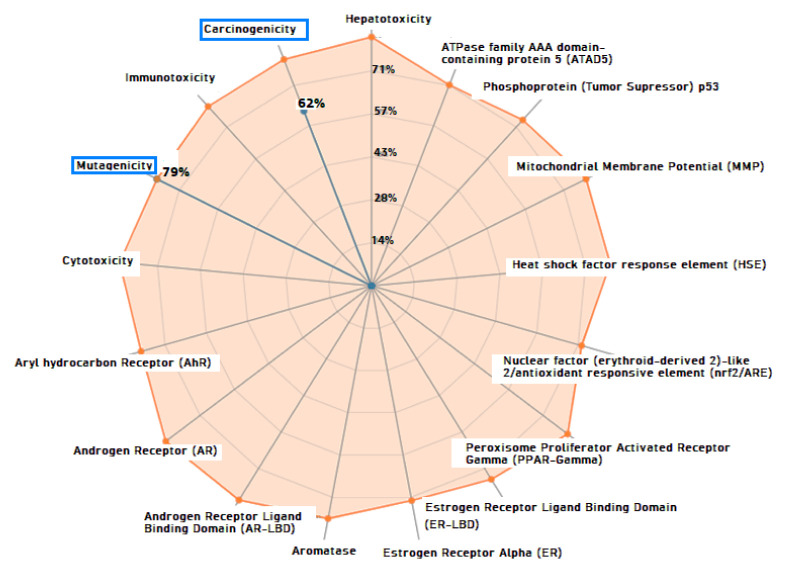
Representation of the toxicity results with the radar chart.

We examined some essential physical and biological parameters in medicinal chemistry using the SwissADME [40] web tool developed by the Swiss Institute of Bioinformatics. These parameters compose of six sections (Table 3), including physicochemical properties, lipophilicity, solubility, pharmacokinetics, drug-likeness, and medicinal chemistry. Druggability predictions (Figure 10) of the title compound to determine the biological targets were performed using SwissTargetPrediction [64]. In Table 3, the pink-colored area of the polygon on the left shows the suitable physicochemical space for oral bioavailability, the white area shows the unsuitable space, and the red lines sign out the position of our compound in the whole space. This hexagon is related to lipophilicity (LIPO), size (SIZE), polarity (POLAR), solubility (INSOLU), unsaturation (INSATU), and flexibility (FLEX) and is calculated by using appropriate domain borders of parameters, including XLOGP, molecular weight, topological polar surface area (TPSA), Log S, fraction of sp^3^, and number of rotatable bonds, respectively. As it can see from the red outline of the polygon, our compound has settled in the range suitable for oral bioavailability. When the BOILED-Egg model of the title molecule is examined from Figure 11, it can see that the molecule has a good intestinal absorption and can cross the blood-brain barrier. Cytochrome P450 enzyme families (CYPs) influence the pharmacokinetics of a drug. We investigated 5 CYPs, including CYP1A2, CYP2C19, CYP2C9, CYP2C6, and CYP3A4, which are related to the 80% of the metabolism of drugs in clinical use [65]. According to the predictions, the title compound is a potential CYPs inhibitor for five CYPs. This inhibitory effect means that our compound as a drug molecule candidate suppresses CYPs enzyme activity and decreases the metabolic rate in human liver, so the pharmacokinetic properties might not reach the desired efficiency.

Drug-likeness was derived from the structures and properties of existing drugs and drug candidates. Before drug discovery, it is important to filter out unsuitable compounds [66]. This term was defined by Lipinski as meeting some proposed criteria for drug candidates [67]. Lipinski states that poor absorption and permeation are more likely in the following situations: i) Molecular weight is higher than 500. ii) LogP value is higher than 5. iii) Hydrogen bond acceptors are higher than 10. iv) Hydrogen bond donors are higher than 5 [68]. After Lipinski, different rules for drug-likeness were given by Ghose (160 ≤ MW ≤ 480; −0.4 ≤ WLOGP ≤ 5.6; −40 ≤ MR ≤ 130; 20 ≤ atoms ≤ 70) [69]; Veber (Rotatable bonds ≤ 10; TPSA ≤ 140) [70]; Egan (WLOGP ≤ 5.88; TPSA ≤ 131.6) [71]; Muegge (200 ≤ MW ≤ 600; −2 ≤ WLOGP ≤ 5; TPSA ≤ 150; number of rings ≤ 7; number of carbons > 4; number of heteroatoms >1; rotatable bonds ≤ 15; hydrogen bond acceptor ≤ 10; hydrogen bond donor ≤ 5) [72]. According to the mentioned rules, our molecule does not violate the above drug-likeness rules. We also investigated druggability predictions for our compound. The calculation results (Figure 10) show that our compound can inhibit the following enzyme classes in the top 15 lists: protease, kinase, phosphodiesterase, family A G protein couplet-receptor, oxidoreductase, voltage-gated ion channel, and cytochrome P450.

### 3.7. Potential multitarget identification with fingerprint methods

To improve the information about the bioactivity properties of our compound, we used a web server, the polypharmacology browser (PPB), www.gdb.unibe.ch. This web server is used to identify potential targets of a compound based on six different fingerprints and some combinations. The results of PPB are given according to the various algorithms: atom pair fingerprint (APfp), extended atom pair fingerprint (Xfp), molecular quantum numbers (MQN), scalar fingerprint counting the occurrence of characters in SMILES (SMIfp), (SMIfp), substructure fingerprint (Sfp), and extended connectivity fingerprint (ECfp4). APfp works with molecular shape; Xfp perceives pharmacophores; MQN perceives atoms, bonds, polarity, ring features, constitution, topology, and molecular shape; SMIfp uses rings, aromaticity, and polarity; Sfp works with the detailed substructures; ECfp4 uses the combination of detailed substructures and pharmacophores [41]. We tabulated the top 20 targets selected by the six fingerprinting algorithms for our compound. The results were ordered by the calculated cumulative density (p-values) for each target in Table 4. The red hexagon in this table indicates that the specified fingerprint algorithm did not find a target; the green hexagon indicates a lower p-value (from 0.01 to 0) and a lower probability for the target; the blue hexagon indicates potential targets for which the p-value is greater than 0.01. Provided that the estimated p-value is greater than 0.01, we indicate the exact p-values. The ChEMBL-ID and common names of the targets, and the explanation of each target are listed in Table 4. The results show that our compound has a similar fingerprint to the molecules with the indicated number on the right side of Table 4, which have strong biological activity on the indicated targets in the ChEMBL database. These targets can cause various diseases, such as malaria: Plasmodium falciparum/ChEMBL364 [73]; cancer: EHMT2/ChEMBL6032 [74]: GMNN/ChEMBL1293278 [75], ALD1AH1/ChEMBL3577 [76]; diabetes: HLP1R/ChEMBL1784 [77]; multiple sclerosis: RORC/ChEMBL1293231 [78, 79], Alzheimer’s disease: MAPT/ChEMBL1293224 [80]; spinocerebellar ataxia: ChEMBL1795085/ATXN2 [81]; HIV-1 infection: APOBEC3G/ChEMBL1741217 [82], APOBEC3F/ChEMBL2007626 [83]; laminopathies: LMNA/ChEMBL1293235 [84]; glioblastomas, chondrosarcomas, and acute myeloid leukemias (AML): IDH1/ChEMBL2007625 [85, 86]; LDL-derived cholesterol, Nieamann-Pick disease type C, and Ebola virus infection: NPC1/ChEMBL1293277 [87]; liver cancer: RAB9A/ChEMBL1293294 [88]. Our compound could interact with the listed targets as a potential ligand molecule and act as an inhibitor against target-related diseases mentioned above.

### 3.8 Toxicity analysis

Two web servers, ProTox-II and pkCSM were used to determine the toxicity parameters of the title compound. The calculated toxicity endpoints and models are shown in Table 5 and Figure 11 (ProTox-II) and Table 6 (pkCSM). From Table 5 and Figure 11, we can see that the title compound is classified as mutagenic and carcinogenic with a probability of 79% and 61%, respectively. From Table 6, we can see that the title compound has two alerts related to AMES mutagenicity and hepatoxicity. In summary, we can define the title compound as mutagenic, carcinogenic, and hepatotoxic. These toxic effects are generally reported as structural warnings for compounds with the nitro substituent [89–92]. Despite these known facts for the toxicophoric nitro groups, many drugs containing the nitro group, such as flutamide and niclosamide, have been approved by the FDA, and the nitro group plays a direct role in the efficacy of a drug molecule [92]; therefore, we cannot exclude the compounds containing the nitro group, and can still consider them as drug candidates.

### 3.9. Gastrointestinal absorption and brain penetration

**Figure 12:**
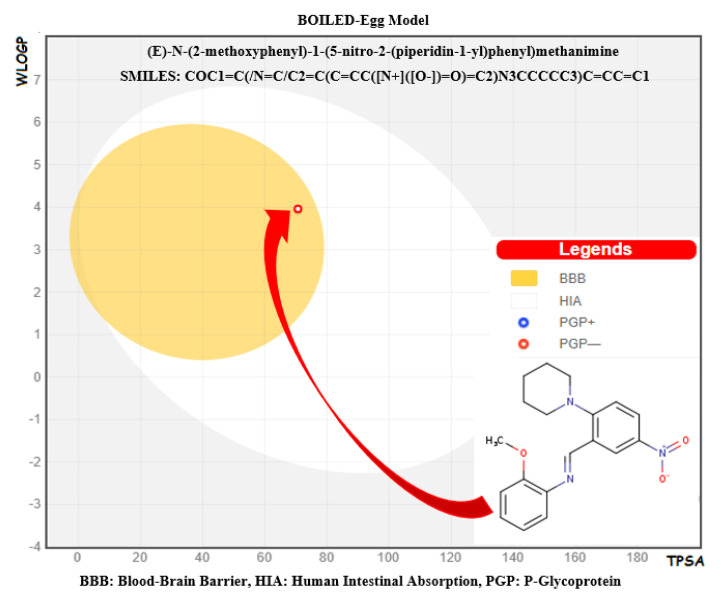
BOILED-Egg Model of query compound: the red cycle in the yolk signs out both good gastrointestinal absorption and blood-brain barrier permeability.

We examined the title compound to determine human intestinal absorption (HIA) and blood-brain barrier (BBB) penetration, two crucial pharmacokinetic properties in drug discovery. These properties were investigated using the Brain Or IntestinaL EstimateD permeation method (BOILED-Egg) developed by Daina and Zoete [93]. This model uses two physicochemical parameters, WLOGP and TPSA. It simultaneously predicts the intestinal absorption and brain access of the molecules. For our compound, the estimated model is shown in Figure 12, in which the yellow area (yolk) shows that the compounds can passively penetrate through the blood-brain barrier. The white region signifies a physicochemical space where the gastrointestinal system can absorb the molecules. In this graph, the white and yellow areas are not mutually exclusive. The small red cycle in the yolk shows that our compound can passively cross the blood-brain barrier and be absorbed by the human gastrointestinal tract. As a result, the molecule is active in the BBB, and the gastrointestinal tract can absorb it.

## 4. Conclusion

A Schiff base compound was synthesized via a condensation reaction between an aromatic aldehyde and amine molecule. The single crystal was analyzed using the X-ray diffraction method. The mentioned compound has space group P21/n and crystallized in monoclinic system. The monomeric units are four in the unit cell (Z = 4).

- The molecular electrostatic potential map and Mulliken charges have revealed the most positive and the most negative regions of the molecule. The oxygen (O3) of the anisole ring and the nitrogen (N3) in the imine group are the most negatively charged atoms. The oxygen atoms (O1 and O2) of the nitro substituent are open positions for the electrophilic attack.
- Molecular orbital analysis provided information on the intramolecular charge transfer, molecular softness, stability, reactivity, and toxicity. The energies of the energy gap, HOMO, and LUMO orbitals were calculated to be −5.629, −2.053, and 3.576 eV, respectively. As a result, charge transfer between the HOMO and LUMO orbitals occurs easily; the title compound has high chemical reactivity, biological activity, polarizability, probably high toxicity, and low kinetic and chemical stability.
- Molecular stability is mainly established by H···H interaction, followed by O···H, C···H, C···C and N···H interactions and others.
- Docking experiments were performed to determine the inhibitory effect of the candidate molecule. The title compound and reference inhibitor were docked to the COVID-19 main protease (Mpro). Our docking calculations showed that the binding energy of the complex of query compound/SARS-CoV-2 (−8.10 kcal/mol) is higher than that of the complex of N3/SARS-CoV-2 (− 7.11 kcal/mol). Therefore, the title compound is a potent candidate for inhibition of the main protease.
- The title compound settled in the suit drug domain region according to the SwissADME algorithm and obeyed the known drug-likeness rules (Lipinski, Veber, Ghose, Egan, Muegge). pkCSM and ProTox-II tools uncovered mutagenic, carcinogenic, and hepatotoxic predictions on the title compound. Metabolism of the molecule in liver is likely to be low, as it was found to be an inhibitor of 5CYPs. There is no concern regarding human intestinal absorption and brain permeability.

## Supplementary information

**Table S1.** Single-crystal X-ray data of the title compound and the refinement parameters.

Crystal data	
CCDC deposition number	2082426
Chemical formula	C _19_H _21_N _3_O _3_
Formula weight	339.39
Temperature (K)	296
Wavelength (Å)	0.71073
Crystal system	Monoclinic
Space group	P2 _1_/n
Unit cell parameters	
a≠b≠c (Å)	10.1685(16), 13.3628(15), 13.416(2)
α=γ≠β (°)	90.00, 90.00, 107.854 (12)
Crystal size (mm)	0.71 × 0.48 × 0.24
Z	4
Volume, V (Å ^3^)	1735.2(4)
μ (mm ^−1^)	0.09
F (000)	720
θ min-max (°)	12.6–35.1
Calculated density (Mgm ^−3^)	1.299
Color and shape	Yellow, prism
Data collection	
Diffractometer	STOE IPDS 2
θ min-max for data collection (°)	2.6–26.0
Index ranges; h, k, l	−10→12, −13→16, −16→16
Measurement method	Scans
Reflections collected	9358
Independent reflections	3398
Reflections with I> 2σ(I)	2080
Absorption correction	Integration ((X-RED32; Stoe and Cie, 2002)
T _min-max_	0.948–0.979
R _int_	0.049
Refinement	
Refinement method	Full matrix least squares on F ^2^
Parameters	227
*R* [ *F* ^2^ > 2σ( *F* ^2^)]	0.041
*wR*( *F* ^2^)	0.094
GooF=S	0.91
Δρ _min-max_ (e Å ^−3^)	−0.13, 0.14
w = 1/[σ ^2^( *F* _o_ ^2^) + (0.0638 *P*) ^2^ + 0.3411 *P*], where P = (Fo2 + 2Fc2)/3, (Δ/σ)max < 0.001,	
Extinction correction: SHELXL-2014/7 (Sheldrick 2014).	

**Table S2.** The calculated and measured bond length values of the title compound.

Bond length	B3LYP	Exp.
O3—C18	1.3713	1.382 (2)
O3—C19	1.4213	1.429 (2)
N2—C5	1.4083	1.406 (2)
N2—C8	1.4673	1.473 (2)
N2—C12	1.4783	1.479 (2)
N3—C7	1.2824	1.277 (2)
N3—C13	1.4006	1.418 (2)
O1—N1	1.2316	1.235 (2)
N1—O2	1.2337	1.230 (2)
N1—C2	1.4647	1.465 (2)
C7—C6	1.4747	1.480 (2)
C7—H7	1.0908	0.9300
C6—C1	1.3989	1.396 (2)
C6—C5	1.425	1.427 (2)
C5—C4	1.4108	1.412 (2)
C13—C14	1.4032	1.404 (2)
C13—C18	1.4214	1.414 (2)
C14—C15	1.3919	1.380 (3)
C14—H14	1.0851	0.9300
C2—C3	1.3954	1.384 (3)
C2—C1	1.3876	1.385 (2)
C18—C17	1.3983	1.390 (3)
C12—C11	1.5304	1.517 (3)
C12—H12A	1.1055	0.9700
C12—H12B	1.0908	0.9700
C1—H1	1.0823	0.9300
C4—C3	1.3868	1.378 (3)
C4—H4	1.0828	0.9300
C3—H3A	1.0827	0.9300
C17—C16	1.3965	1.389 (3)
C17—H17	1.0832	0.9300
C8—C9	1.532	1.517 (3)
C8—H8A	1.0834	0.9700
C8—H8B	1.1062	0.9700
C11—C10	1.5337	1.529 (3)
C11—H11A	1.0966	0.9700
C11—H11B	1.0968	0.9700
C15—C16	1.3928	1.379 (3)
C15—H15	1.0852	0.9300
C16—H16	1.0858	0.9300
C9—C10	1.5329	1.521 (3)
C9—H9A	1.0968	0.9700
C9—H9B	1.096	0.9700
C10—H10A	1.0956	0.9700
C10—H10B	1.0991	0.9700
C19—H19A	1.0972	0.9600
C19—H19B	1.0966	0.9600

**Table S3.** The calculated and measured bond angle values of the title compound.

Bond angle	B3LYP	Exp.
C18—O3—C19	117.9116	117.61 (15)
C5—N2—C8	117.3559	117.57 (14)
C5—N2—C12	116.3752	117.31 (13)
C8—N2—C12	112.0595	111.20 (13)
C7—N3—C13	123.512	123.96 (15)
O2—N1—O1	124.406	122.87 (18)
O2—N1—C2	117.6418	118.15 (19)
O1—N1—C2	117.9521	118.96 (16)
N3—C7—C6	120.3003	119.71 (15)
N3—C7—H7	123.2613	120.1
C6—C7—H7	116.3552	120.1
C1—C6—C5	119.3966	119.37 (16)
C1—C6—C7	118.9256	117.49 (15)
C5—C6—C7	121.5157	122.78 (15)
N2—C5—C4	121.3611	120.96 (16)
N2—C5—C6	120.0819	120.74 (15)
C4—C5—C6	118.5476	118.25 (17)
C14—C13—C18	118.0087	117.35 (16)
C14—C13—N3	115.9861	115.56 (15)
C18—C13—N3	125.8697	127.02 (15)
C15—C14—C13	122.0243	122.75 (17)
C15—C14—H14	121.0932	118.6
C13—C14—H14	116.8814	118.6
C3—C2—C1	121.3513	121.30 (17)
C3—C2—N1	119.1791	119.95 (17)
C1—C2—N1	119.4628	118.71 (17)
O3—C18—C17	123.0735	123.13 (16)
O3—C18—C13	117.1253	116.91 (15)
C17—C18—C13	119.7842	119.93 (17)
N2—C12—C11	111.0694	110.75 (14)
N2—C12—H12A	109.4321	109.5
C11—C12—H12A	109.5931	109.5
N2—C12—H12B	109.1818	109.5
C11—C12—H12B	110.2807	109.5
H12A—C12—H12B	107.1988	108.1
C2—C1—C6	120.2402	120.23 (17)
C2—C1—H1	120.6299	119.9
C6—C1—H1	119.0941	119.9
C3—C4—C5	121.5246	121.42 (18)
C3—C4—H4	118.6577	119.3
C5—C4—H4	119.7861	119.3
C4—C3—C2	118.8524	119.43 (17)
C4—C3—H3A	121.5638	120.3
C2—C3—H3A	119.5709	120.3
C16—C17—C18	120.6792	120.48 (18)
C16—C17—H17	119.2051	119.8
C18—C17—H17	120.113	119.8
N2—C8—C9	110.5135	110.87 (16)
N2—C8—H8A	108.377	109.5
C9—C8—H8A	109.9995	109.5
N2—C8—H8B	111.3825	109.5
C9—C8—H8B	109.0347	109.5
H8A—C8—H8B	107.4803	108.1
C12—C11—C10	111.2726	110.75 (17)
C12—C11—H11A	108.8787	109.5
C10—C11—H11A	110.5794	109.5
C12—C11—H11B	108.756	109.5
C10—C11—H11B	109.8197	109.5
H11A—C11—H11B	107.4352	108.1
C16—C15—C14	119.259	118.63 (18)
C16—C15—H15	120.6244	120.7
C14—C15—H15	120.1082	120.7
C15—C16—C17	120.185	120.83 (18)
C15—C16—H16	120.5368	119.6
C17—C16—H16	119.2749	119.6
C8—C9—C10	111.096	111.76 (15)
C8—C9—H9A	108.7378	109.3
C10—C9—H9A	109.829	109.3
C8—C9—H9B	108.9651	109.3
C10—C9—H9B	110.8098	109.3
H9A—C9—H9B	107.3022	107.9
C9—C10—C11	110.1514	109.04 (16)
C9—C10—H10A	110.6369	109.9
C11—C10—H10A	110.646	109.9
C9—C10—H10B	109.3121	109.9
C11—C10—H10B	109.3268	109.9
H10A—C10—H10B	106.691	108.3
O3—C19—H19A	111.7356	109.5
O3—C19—H19B	111.4225	109.5
H19A—C19—H19B	109.1408	109.5
O3—C19—H19C	106.0313	109.5
H19A—C19—H19C	108.9719	109.5
H19B—C19—H19C	109.457	109.5

**Table S4.** The calculated and measured dihedral angle values of the title compound.

Dihedral angle	B3LYP	Exp.
C13—N3—C7—C6	−177.4069	−173.59 (14)
N3—C7—C6—C1	11.9856	23.1 (2)
N3—C7—C6—C5	−172.6713	−163.85 (14)
C8—N2—C5—C4	−21.2475	14.0 (2)
C12—N2—C5—C4	−115.5165	−122.78 (17)
C8—N2—C5—C6	−157.6223	−163.30 (14)
C12—N2—C5—C6	65.6137	59.93 (19)
C1—C6—C5—N2	−177.6607	178.00 (13)
C7—C6—C5—N2	7.0177	5.1 (2)
C1—C6—C5—C4	3.4379	0.6 (2)
C7—C6—C5—C4	−171.8837	−172.28 (14)
C7—N3—C13—C14	−146.5829	150.97 (16)
C7—N3—C13—C18	−37.755	−32.2 (2)
C18—C13—C14—C15	2.8046	1.4 (2)
N3—C13—C14—C15	178.8237	178.52 (15)
O2—N1—C2—C3	−0.6199	−7.2 (2)
O1—N1—C2—C3	179.2988	171.88 (17)
O2—N1—C2—C1	178.4461	170.37 (16)
O1—N1—C2—C1	−1.6352	−10.5 (2)
C19—O3—C18—C17	10.6209	−3.2 (3)
C19—O3—C18—C13	−167.8739	178.78 (16)
C14—C13—C18—O3	177.2472	177.67 (14)
N3—C13—C18—O3	1.6641	0.9 (2)
C14—C13—C18—C17	−1.2993	−0.5 (2)
N3—C13—C18—C17	−176.8826	−177.24 (16)
C5—N2—C12—C11	−162.6825	−161.74 (15)
C8—N2—C12—C11	−58.3476	58.9 (2)
C3—C2—C1—C6	−0.4093	0.1 (2)
N1—C2—C1—C6	−179.4544	−177.44 (14)
C5—C6—C1—C2	−2.1637	−0.6 (2)
C7—C6—C1—C2	173.2798	172.71 (14)
N2—C5—C4—C3	178.8666	−177.59 (15)
C6—C5—C4—C3	−2.2467	−0.2 (2)
C5—C4—C3—C2	−0.2714	−0.2 (2)
C1—C2—C3—C4	1.6429	0.3 (2)
N1—C2—C3—C4	−179.3094	177.82 (14)
O3—C18—C17—C16	−179.1932	−179.03 (17)
C13—C18—C17—C16	−0.7367	−1.0 (3)
C5—N2—C8—C9	162.3447	163.00 (14)
C12—N2—C8—C9	−59.1211	−57.74 (19)
N2—C12—C11—C10	−54.8559	−57.9 (2)
C13—C14—C15—C16	−2.2182	−0.7 (3)
C14—C15—C16—C17	0.0843	−0.8 (3)
C18—C17—C16—C15	1.3698	1.7 (3)
N2—C8—C9—C10	56.8131	56.3 (2)
C8—C9—C10—C11	−53.6878	−54.6 (2)
C12—C11—C10—C9	52.7361	55.2 (2)

Figure S1. FTIR spectrum of the title compound.

Figure S2. UV-Vis spectrum of the title compound.

Figure S3. Grid box including the active residues in Mpro of SARS-CoV-2.

Figure S4. The comparative docking results of reference inhibitor N3 and query compound.

## Figures and Tables

**Table 1:** Calculated global reactivity descriptors.

Parameters	Values (eV)
E_HOMO_	−5.629
E_LUMO_	−2.053
Energy band gap (ΔE = E_LUMO_−E_HOMO_)	3.576
Ionization potential (I = − E_HOMO_)	5.629
Electron affinity (A = − E_LUMO_)	2.053
Chemical hardness (η = (I−A)/2)	1.788
Chemical softness (σ = 1/2η)	0.279
Electronegativity (χ = (I+A)/2)	3.841
Chemical potential (μ = −(I+A)/2)	−3.841
Electrophilicity index (ω = μ^2^/2η)	4.125
Maximum charge transfer index (ΔN_max_ = −μ/η)	2.148

**Table 2:** Comparative docking studies performed with Mpro (3CLpro) of SARS-CoV-2.

	PDB ID	Compound class	Compound code with the highest docking score	Common groups with the title compound	Software	Docking score (kcal/mol)	Interaction with the catalytic dyad
Our study (S0)	6LU7	Schiff base	Title compound	Aromatic ring, alicyclic ring, imine group, nitro group, methoxy group, and tertiary amine	AutoDock	−8.10	No
S1 [94]	6LU7	Coumarin derivatives	10a	Two aromatic rings	Autodock and AutoDock Vina	−7.0 and −8.6	Yes
S2 [95]	7BQY	Coumarin derivatives	14	Aromatic ring and imine group	MOE	−7.47	Not reported
S3 [96]	6LU7	Crinipellin and Alliacol-B derivatives	Compound 5	One alicyclic ring	AutoDock	−7.3	No
S4 [97]	6LU7	Azo-imidazole derivatives	L5	Methoxy, two aromatic rings, imine group	AutoDock Vina	−8.1	Yes
S5 [98]	7BQY	Schiff bases	ZG-7	Not reported	MOE	−8.79	Not reported

**Table 3:** Some calculated drug-like properties of the title compound.

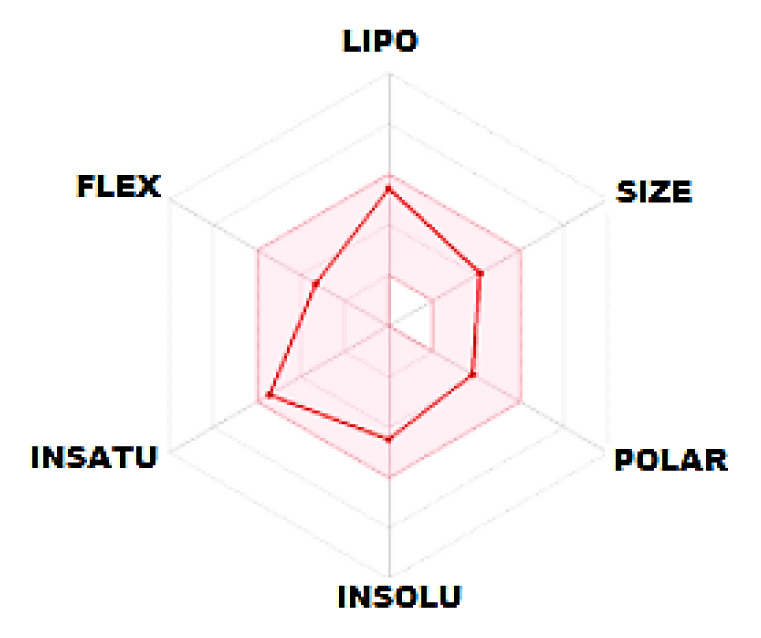		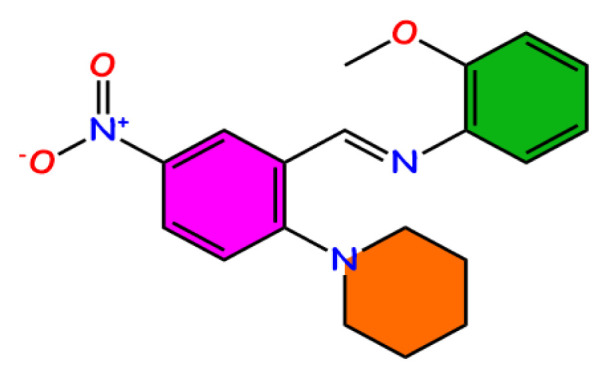	
Physicochemical properties		Pharmacokinetics	
Formula	C_19_H_21_N_3_O_3_	GI absorption	High
Molecular weight	339.39	BBB permeant	Yes
Num. heavy atoms	25	P-gp substrate	No
Num. arom. heavy atoms	12	CYP1A2 inhibitor	Yes
Fraction Csp^3^	0.32	CYP2C19 inhibitor	Yes
Num. Rotatable bonds	5	CYP2C9 inhibitor	Yes
H-bond acceptors	4	CYP2D6 inhibitor	Yes
H-bond donors	0	CYP3A4 inhibitor	Yes
Molar reactivity	104.90	Log K_p_ (skin permeation)	−5.54 cm/s
TPSA	70.65 Å^2^		
**Lipophilicity**		**Drug-likeness**	
Log P_o/w_ (İLOGP)	3.05	Lipinski	Yes, 0 violation
Log P_o/w_ (XLOGP)	3.99	Ghose	Yes
Log P_o/w_ (WLOGP)	3.96	Veber	Yes
Log P_o/w_ (MLOGP)	2.07	Egan	Yes
Log P_o/w_ (SILICOS_IT)	2.29	Muegge	Yes
Consensus Log P_o/w_	3.07	Bioavailability score	0.55
**Medicinal chemistry**			
PAINS	1 alert: anil_di_alk_A		
Brenk	3 alerts: imine_1, nitro_group, oxygen-nitrogen_single bond		
Leadlikeness	No, 1 violation: XLOGP > 3.5		
Synthetic accessibility	2.98		
**Water solubility**			
Log *S* (ESOL)	−4.48		
Solubility	1.12 × E-02 mg/mL; 3.29 × E-05 mol/L		
Class	Moderately soluble		
Log S (Ali)	−5.18		
Solubility	2.27 × E-03 mg/mL; 6.68 × E-06 mol/L		
Class	Moderately soluble		
Log S (SILICOS-IT)	−5.21		
Solubility	2.11 × E-03 mg/mL; 6.21 × E-06 mol/L		
Class	Moderately soluble		

**Table 4:** The potential targets of the title compound calculated by PPB.

Rank	ChEMBL-ID	ChEMBL-name	APfp	Xfp	MQN	SMIfp	Sfp	ECfp4	No of mols
1	ChEMBL364	ORGANISM_NOGN	 0.233	 0.113	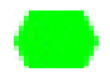	 0.032	 0.236	 0.036	16
2	ChEMBL6032	EHMT2	 0.533	 0.114	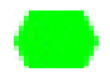	 0.117	 0.233	 0.025	11
3	ChEMBL1784	GLP1R		 0.109	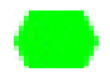	 0.030	 0.207	 0.021	17
4	ChEMBL1293278	GMNN		 0.119		 0.032	 0.234	 0.013	19
5	ChEMBL1293231	RORC	 0.264	 0.109		 0.105		 0.065	6
6	ChEMBL1741220	BAZ2B		 0.241		 0.06	 0.232	 0.013	5
7	ChEMBL1293224	MAPT	 0.504		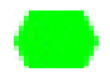	 0.083	 0.160	 0.018	9
8	ChEMBL1795085	ATXN2		 0.301	 0.262	 0.108	 0.297	 18	12
9	ChEMBL1741209	ATAD5	 0.523		 0.012			 0.028	12
10	ChEMBL614818	CELL-LINE_NOGN	 0.556	 0.121		 0.032			11
11	ChEMBL1741217	APOBEC3G		 0.079				 0.012	13
12	ChEMBL1293235	LMNA				 0.092		 0.029	8
13	ChEMBL4377	GNAS		 0.111		 0.035		 0.030	5
14	ChEMBL614358	CELL-LINE_NOGN		 0.018					11
15	ChEMBL1293258	SMAD3			 0.011				2
16	ChEMBL2007625	IDH1		 0.109					12
17	ChEMBL3577	ALDH1A1		 0.264			 0.199		3
18	ChEMBL1293277	NPC1	 0.439	 0.234	 0.054	 0.078		 0.081	4
19	ChEMBL1293294	RAB9A	 0.465	 0.286	 0.059			 0.083	4
20	ChEMBL2007626	APOBEC3F							6
	Target not found by fingerprint.								
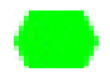	p-value stretching from 0.01 to 0.								
	p-value > 0.01.								

ORGANISM_NOGN: Plasmodium falciparum; EHMT2: Histone-lysine_N-methyltransferase_H3_lysine-9_specific_3; GLP1R: Glucagon-like_peptide_1_receptor; GMNN: Geminin; RORC: Nuclear_receptor_ROR-gamma; BAZ2B: Bromodomain_adjacent_to_zinc_finger_domain_protein_2B; MAPT: Microtubule-associated_protein_tau; ATXN2: Ataxin-2; ATAD5: ATPase_family_AAA_domain-containing_protein_5; CELL-LINE_NOGN: HEK293; APOBEC3G: DNA_dC->dU-editing_enzyme_APOBEC-3G; LMNA: Prelamin-A/C; GNAS: Guanine_nucleotide-binding_protein_G(s)_subunit_alpha; CELL-LINE_NOGN: BJ; SMAD3: Mothers against decapentaplegic homolog_3; IDH1: Isocitrate_dehydrogenase_[NADP]_cytoplasmic; ALDH1A1: Aldehyde_dehydrogenase_1A1; NPC1: Niemann-Pick_C1_protein; RAB9A: Ras-related_protein_Rab-9A; APOBEC3F: DNA_dC->dU-editing_enzyme_APOBEC-3F.

**Table 5:** Toxicity results of the title compound calculated by ProTox-II.

Toxicity model report				
Classification	Target	Shorthand	Prediction	Probability
Organ toxicity	Hepatotoxicity	dili	Inactive	0.68
Toxicity end points	Carcinogenicity	carcino	Active	0.61
Toxicity end points	Immunotoxicity	immuno	Inactive	0.82
Toxicity end points	Mutagenicity	mutagen	Active	0.79
Toxicity end points	Cytotoxicity	cyto	Inactive	0.60
Tox21-Nuclear receptor signaling pathways	Aryl hydrocarbon receptor (AhR)	nr_ahr	Inactive	0.69
Tox21-Nuclear receptor signaling pathways	Androgen receptor (AR)	nr_ar	Inactive	0.90
Tox21-Nuclear receptor signaling pathways	Androgen receptor (AR)-ligand binding domain (AR-LBD)	nr_ar_Ibd	Inactive	0.97
Tox21-Nuclear receptor signaling pathways	Aromatase	nr_aromatase	Inactive	0.72
Tox21-Nuclear receptor signaling pathways	Estrogen receptor (ER)	nr_er	Inactive	0.87
Tox21-Nuclear receptor signaling pathways	Estrogen receptor (ER)-ligand binding domain (AR-LBD)	ner_er_Ibd	Inactive	0.98
Tox21-Nuclear receptor signaling pathways	Peroxisome proliferator activated receptor gamma (PPAR-Gamma)	nr_ppar_gamma	Inactive	0.98
Tox21-Stress response pathways	Nuclear factor (erythroid-derived 2)-like2/antioxidant responsive element (nrf2/ARE)	ar_are	Inactive	0.86
Tox21-Stress response pathways	Heat shock factor response element (HSE)	sr_hse	Inactive	0.86
Tox21-Stress response pathways	Mitochondrial membrane potential (MMP)	sr_mmp	Inactive	0.52
Tox21-Stress response pathways	Phosphoprotein (tumor suppressor) p53	sr_p53	Inactive	0.89
Tox21-Stress response pathways	ATPase family AAA domain-containing protein 5 (ATAD5)	sr_atad5	Inactive	0.88

**Table 6:** Some toxicity parameters and results calculated by pkCSM of the title compound

pkCSM toxicity report.		
AMES toxicity	Yes	Categorical (Yes/No)
Max. tolerated dose (human)	0.076	Numeric (log mg/kg/day)
Herg I inhibitor	No	Categorical (Yes/No)
Herg II inhibitor	No	Categorical (Yes/No)
Oral Rat Acute Toxicity (LD50)	2.549	Numeric (mol/kg)
Oral Rat Chronic Toxicity (LOAEL)	1.383	Numeric (log mg/kg_bw/day)
Hepatotoxicity	Yes	Categorical (Yes/No)
Skin sensitization	No	Categorical (Yes/No)
*T. Pyriformis* toxicity	1.417	Numeric (log ug/L)
Minnow toxicity	−1.982	Numeric (log mM)

## Appendix A Supplementary data

CCDC 2082426 contains the supplementary crystallographic data for the title compound. These data can be obtained free of charge via http://www.ccdc.cam.ac.uk/conts/retrieving.html, or from the Cambridge Crystallographic Data Centre, 12 Union Road, Cambridge CB2 1EZ, UK; fax: (+44) 1223-336-033; or e-mail: deposit@ccdc.cam.ac.uk.
