# Supplementary figures and images for: Synthesis, characterization, computational analyses, in silico ADMET studies, and inhibitory action against SARS-CoV-2 main protease (Mpro) of a Schiff base
Source: Turk J Chem. 2022 Jun 2;46(5):1548–64. doi: 10.55730/1300-0527.3460 (PMC10390206; doi:10.55730/1300-0527.3460)

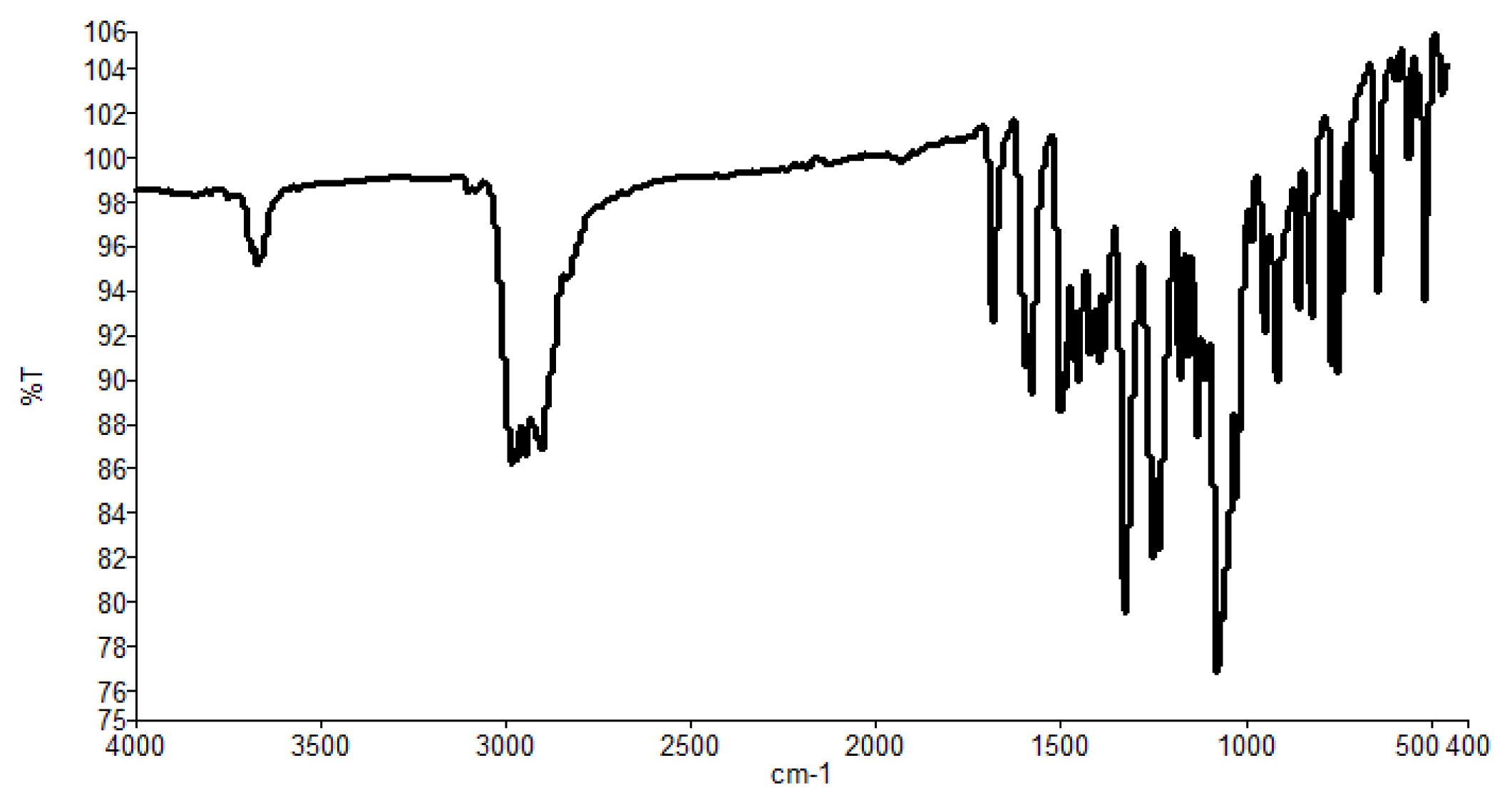

Supplement: Figure S1. — FTIR spectrum of the title compound. [file turkjchem-46-5-1548s1.tif]

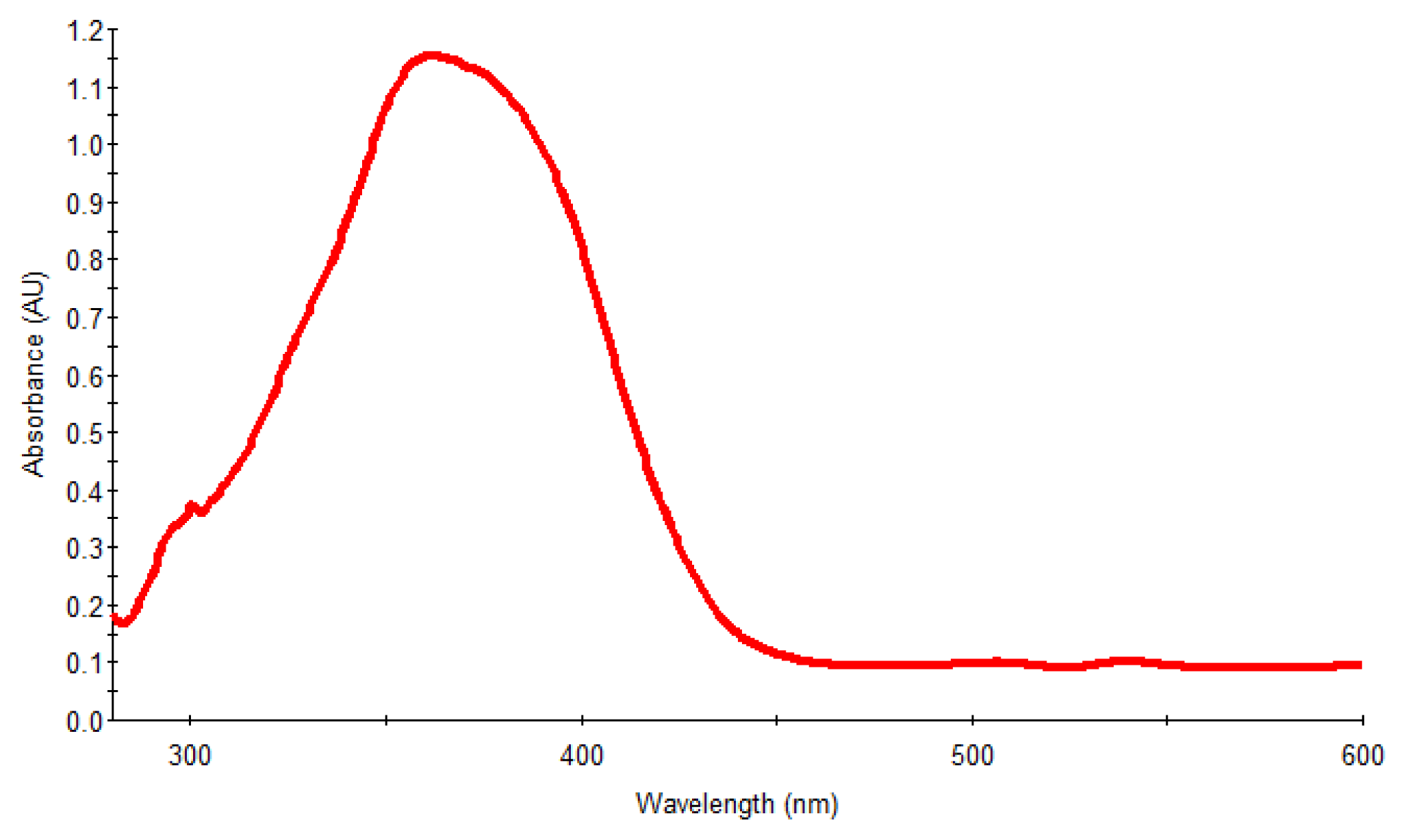

Supplement: Figure S2. — UV-Vis spectrum of the title compound. [file turkjchem-46-5-1548s2.tif]

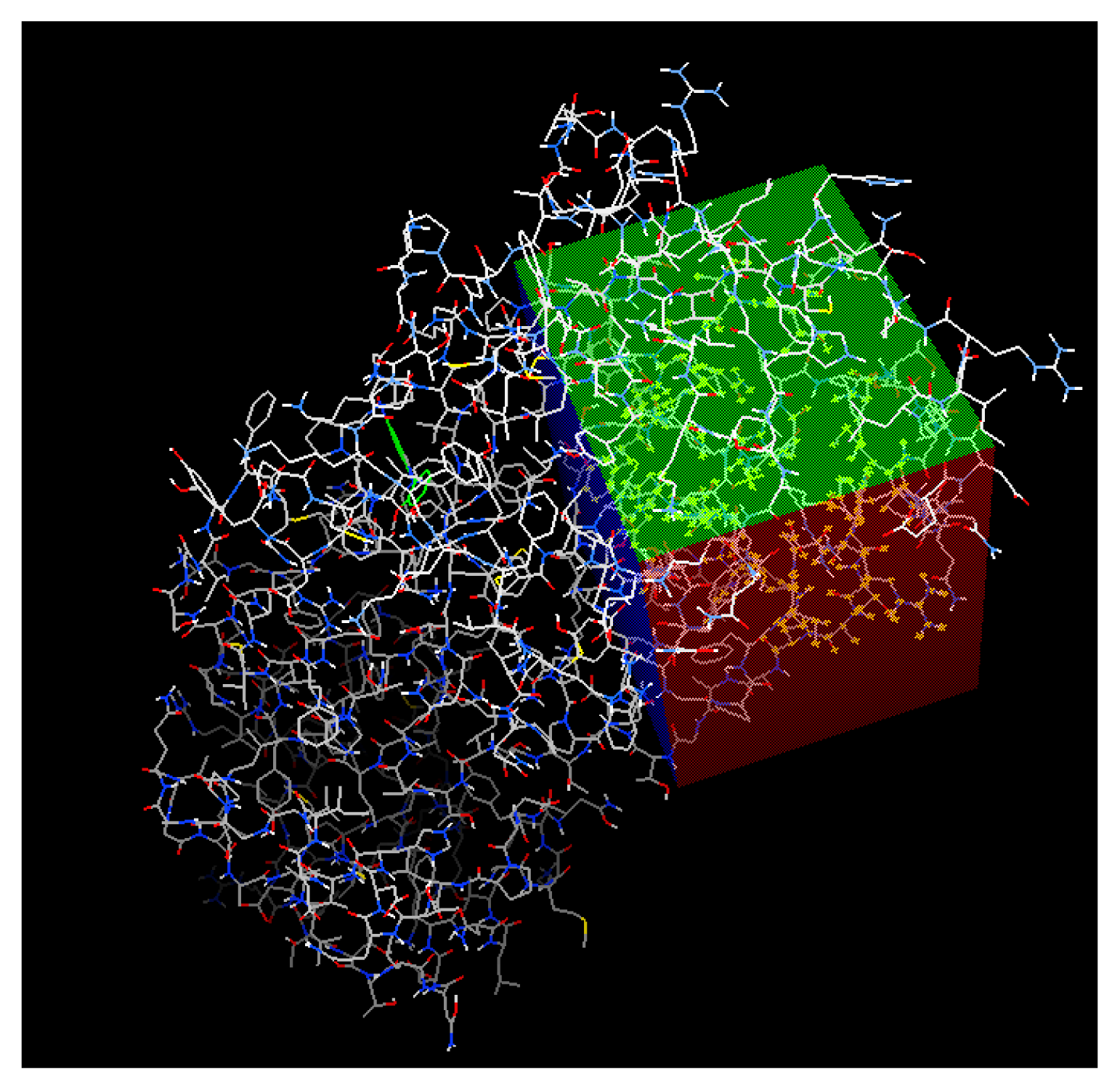

Supplement: Figure S3. — Grid box including the active residues in Mpro of SARS-CoV-2. [file turkjchem-46-5-1548s3.tif]

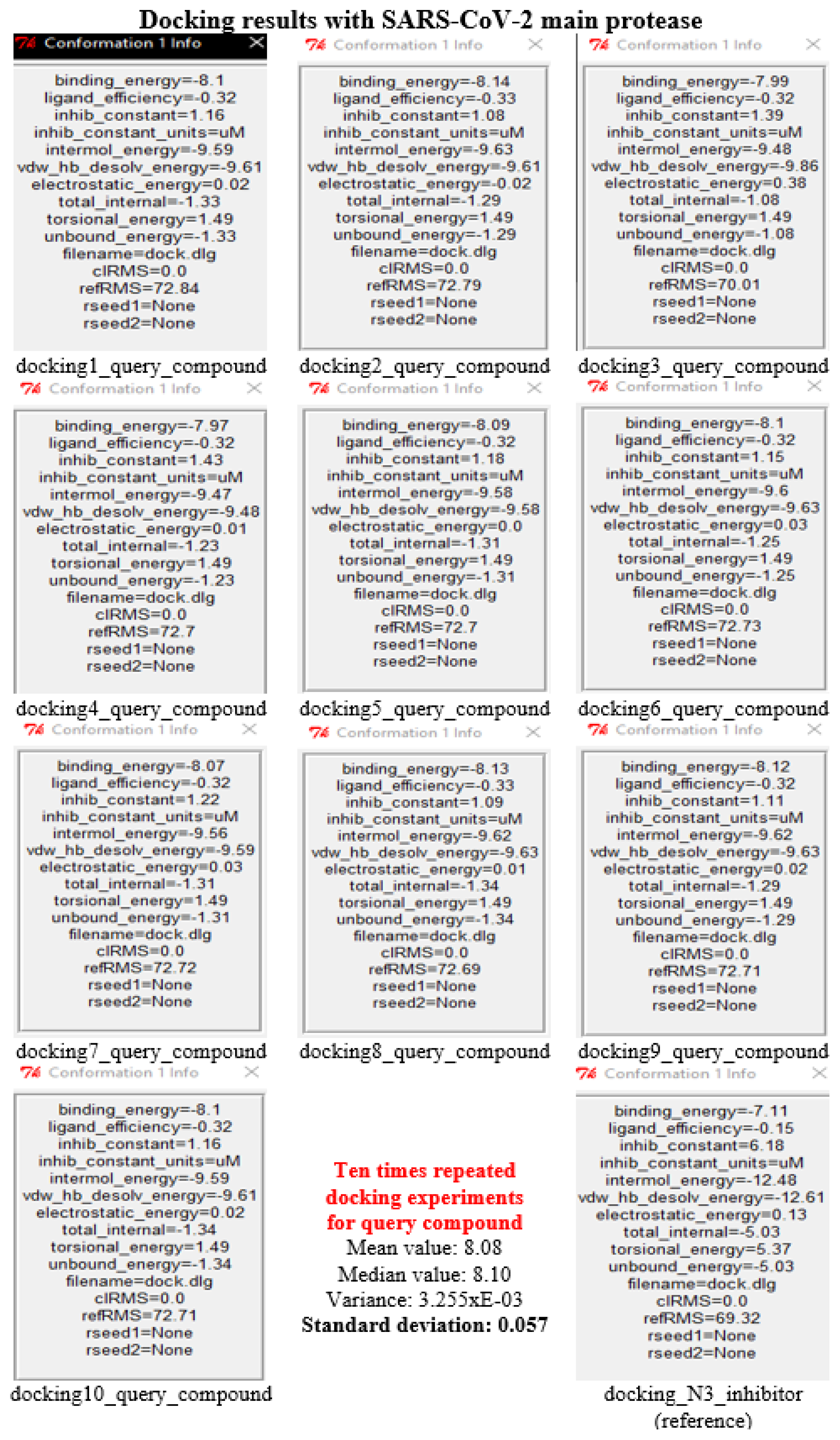

Supplement: Figure S4. — The comparative docking results of reference inhibitor N3 and query compound. [file turkjchem-46-5-1548s4.tif]
